# Application of acidic treated pumice as an adsorbent for the removal of azo dye from aqueous solutions: kinetic, equilibrium and thermodynamic studies

**DOI:** 10.1186/1735-2746-9-9

**Published:** 2012-11-05

**Authors:** Mohammad Reza Samarghandi, Mansur Zarrabi, Mohammad Noori sepehr, Abdeltif Amrane, Gholam Hossein Safari, Saied Bashiri

**Affiliations:** 1 Department of Environmental Health and Research Center for Health Sciences, Hamadan University of Medical Sciences, Hamadan, Iran; 2 Department of Environmental Health Engineering, Faculty of Health, Alborz University of Medical Sciences, Karaj, Iran; 3 Ecole Nationale Supérieure de Chimie de Rennes, CNRS, Avenue du Général Leclerc, Rennes, France; 4 Department of Environmental Health Engineering, Faculty of Health, Tehran University of Medical Sciences, Tehran, Iran; 5 Young Researchers Club, Science and Research Branch, Islamic Azad University, Tehran, Iran

**Keywords:** Adsorption, Equilibrium studies, Pumice, Azo Dye

## Abstract

Colored effluents are one of the important environment pollution sources since they contain unused dye compounds which are toxic and less-biodegradable. In this work removal of Acid Red 14 and Acid Red 18 azo dyes was investigated by acidic treated pumice stone as an efficient adsorbent at various experimental conditions. Removal of dye increased with increase in contact time and initial dye concentration, while decreased for increment in solution temperature and pH. Results of the equilibrium study showed that the removal of AR14 and AR18 followed Freundlich (r^2^>0.99) and Langmuir (r^2^>0.99) isotherm models. Maximum sorption capacities were 3.1 and 29.7 mg/g for AR 14 and AR18, namely significantly higher than those reported in the literature, even for activated carbon. Fitting of experimental data onto kinetic models showed the relevance of the pseudo-second order (r^2^>0.99) and intra-particle diffusion (r^2^>0.98) models for AR14 and AR18, respectively. For both dyes, the values of external mass transfer coefficient decreased for increasing initial dye concentrations, showing increasing external mass transfer resistance at solid/liquid layer. Desorption experiments confirmed the relevance of pumice stone for dye removal, since the pH regeneration method showed 86% and 89% regeneration for AR14 and AR18, respectively.

## Introduction

Dyes are widely used in many industries such as textiles, dyeing, printing, paper, plastic and leather manufactory. Every year about more than 7×105 tons of different commercial dyes and pigments are produced all over the world, and about 65 - 70% of them are classified (included) into azo compounds [1,2]. Colored effluents from textile industries are important environmental pollution sources. Such effluents usually contain non-used dyes, as well as several organic and inorganic pollutants [3,4]. Synthetic dyes, often used in these industries, can be divided into acidic, reactive, direct, basic and other groups.

The Azo dyes have an azo group band (−N=N-) and because of their low cost, variety, solubility and stability are the most common synthetic dyes used for dying [3,5]. It is estimated that azo dyes constitute over 50% of the total dye production [1]. Without appropriate treatment, the release of such wastewater into the environment is a serious problem for aquatic environment and human health. For this reason, the treatment of colored waste is necessary.

Several methods such as aerobic-anaerobic treatment [6], advanced oxidation [7], membrane processes [8] and zeolite materials [9] have been used for dye removal from dyestuff effluent. Adsorption is a key process that is used for the removal of pollutants from wastewater for its low cost, easy application and effectiveness [10]. Commercial activated carbon is the most common adsorbent that has been implemented in this process. This product is very effective for removal of pollutants but is expensive [4]. Recently several natural materials such as eggshell and chitosan [11,12], oxihumolite [13] and flay ash [14] have been tested as sorbents. Among these natural materials, pumice which is a volcanic stone has a low weight and a porous structure (up to 85%) and can be found in many regions of the world. Because of its micro-porous structure, pumice has a high specific surface area, which is advantageous since it allows avoiding the preliminary step of calcinations, a high energy cost, and can float in water owing to its low density.

Recently, many researchers have used pumice for removal of cadmium [15], disinfection by-product [16], heavy metals [17] and sulfur dioxide [18]. Due to the several advantages of pumice stone and its accessibility in Iran, the aim of the present work was to investigate its effectiveness for the removal of two azo dyes, Acid Red 14 (AR 14) and Acid Red 18 (AR 18) at various experimental conditions. Since initial pumice had some impurities, showing low sorption capacity (data are not shown) and are also negatively charged [19], the acidic treated pumice was used in this work. Therefore, the purpose for acidic treatment of pumice was to improve the positive surface charge of adsorbent and improve its sorption capacity.

## Materials and methods

### Preparation and characterization of the sorbent

Pumice stone was obtained from Tikmeh Dash region of eastern Azerbaijan (Iran). The present sorbent was light exhibit and had 85% porosity. The natural pumice was washed several times by double-distillated water and then put in 1 N HCL acid for 24 hours. After that, the pumice sample was taken and rinsed several times by double-distillated water until its effluent turbidity reached at least 0.1 NTU. Finally, the prepared sample was crushed and sieved to 20 meshes and used as sorbent. The chemical structure of the sorbent was determined by X-Ray Fluorescence (Model XRert, Holland) and the results are shown in Table 1. In addition, the morphology of the pumice before and after preparation was characterized by SEM (XL30, Philips).

**Table 1:** **Adsorption characterization based on the *R***_***L***_**values**

**R**_**L**_**value**	**Type of process**
R_L_>1	Unfavorable
R_L_=1	Linear
0<R_L_<1	Favorable
R_L_=0	Irreversible

### Chemicals and experiments

All chemicals used in this work were of GR grade and were obtained from Merck (Germany). All experiments were conducted in batch system and in 250 mL conical flasks. pH was adjusted by 1 N H_2_SO_4_ or NaOH (Sartorius PP-50). Various parameters such as pH (3–11), temperature (20-60°C), initial dye concentration (50–120 mg/L) and contact time were examined. During a given experiment, a specified amount of adsorbent was added to dye solution at given pH and then shaken at 200 rpm (Hanna-Hi 190M, Singapore). At predetermined time intervals, samples were taken, filtered, centrifuged and the dye concentration was spectrophotometrically determined (UV/VIS spectrophotometer, Shimadzo-1700, Japan) as follows:

(1) $$q_{e}=\left(C_{0}-C_{e}\right)V/M\left(1\right)$$

Where q_e_ (mg/g) was the amount of dye adsorbed on pumice, C_0_ and C_e_ were the initial and equilibrium dye concentrations (mg/L), respectively; V (L) was the volume of solution and M (gram) was the mass of pumice.

### Equilibrium study

The design of adsorption process also relies on the kinetic study. The rate of chemical reaction is explained by chemical kinetic [20]. The most common kinetic models for adsorption are pseudo-first order, pseudo-second order and modified pseudo-first order models [21]. Since the equilibrium time needs to be determined prior to experiments, a specified amount of 0.5 g pumice was added to 250 mL beakers. After that, 150 mL of dye solution was transferred into each beaker. The beaker was shaken at an agitation speed of 150 rpm and a constant temperature. At predetermined time intervals, the beakers were removed from the shaker and the dye concentration was spectrophotometrically determined. In this work, four kinetic model namely pseudo-first order model, pseudo-second order model, modified pseudo-first order model and intra-particle diffusion model were studied. The Pseudo-first order kinetic is described by the following equation [21]:

(2) $$\mathrm{Log}\;\left(1/q_{t}1/q_{e}\right)=-\left(k_{1}/2.303\right)t$$

Where q_e_ and q_t_ are the amount of adsorbed dye (mg/g) at equilibrium and at time *t* (min) respectively; and *k*_*1*_ is the rate constant (1/min). Pseudo-second order kinetic is expressed as follows [21]:

(3) $$t/q_{t}=1/\left(k_{2}{q_{2}}^{2}\right)+\left(1/q_{e}\right)t$$

Where k_2_ is the rate constant (g/mg min). The modified pseudo-first order model was proposed by Azizian and Bashiri [20]:

(4) $$\mathrm{Log}\;\left(1-q_{t}/q_{e}\right)+q_{t}/q_{e}=-k_{m}t$$

Due to disability of the all above kinetic models to describe diffusion mechanism, kinetic data can be analyzed by means of intra-particle diffusion model [22]. The overall shape of this model is:

(5) $$q=k_{i}t^{0.5}+C$$

By linear plotting of q vs. t^0.5^, the values of k_i_ and C can be obtained from the slope and the intercept of this plot.

### Isotherm study

The design of an adsorption process relies on equilibrium experiments known as adsorption isotherms. The Langmuir and Freundlich isotherm models are the most commonly used for the description of equilibrium data. Langmuir isotherm model is based on the assumption of a homogenous surface energy distribution. In present work, the equilibrium data were analyzed by linear model of isotherm model. The linear (Eq. (6)) shape of Langmuir model is described as follows:

(6) $$C_{e}/q_{e}=C_{e}/q_{m}+1/\left(q_{m}b\right)$$

Where q_e_ is the equilibrium amount of adsorbate (mg/g), C_e_ is the equilibrium concentration of adsorbate (mg/L), q_m_ is the maximum adsorption capacity and b is the Langmuir constant. The important feature of the Langmuir model can be deduced from analysis of a specific parameter known as R_L_ and expressed as follows:

(7) $$R_{L}=1/\left(1+{\mathrm{bC}}_{0}\right)$$

Adsorption can be characterized based on the *R*_*L*_ value (Table 2).

**Table 2:** Chemical components of the used pumice (w/w)

**Component**	**%**	**Component**	**%**
**SiO**_**2**_	51.45	SrO	0.227
**Al**_**2**_**O**_**3**_	17.08	MnO	0.092
**TiO**_**2**_	1.54	K_2_O	3.26
**P**_**2**_**O**_**5**_	0.661	SO_3_	0.529
**CaO**	6.44	Na_2_O	5.67
**Fe**_**2**_**O**_**3**_	6.32	Specific Surface Area (by BET method)	54m^2^/g
**MgO**	6.17		

The Freundlich model is another common isotherm model, used for heterogeneous systems. This model can be expressed as follows:

(8) $$\log\;\left(q_{e}\right)=\log\;\left(k_{f}\right)+1/\mathrm{n log}\left(C_{e}\right)$$

Where k_f_ and n are the Freundlich constants. A high value of k_f_ characterizes a high affinity of adsorbate. For favorable adsorption, the value of Freundlich constant (n) should be in the range of 1–10. The Temkin isotherm has been used for heterogeneous adsorption of adsorbate on a surface. The following is the linear form of the Temkin model:

(9) $$q_{e}=B_{1}\ln\;\left(k_{t}\right)+B_{1}\ln\;\left(C_{e}\right)$$

Where B_1_=RT/b_1_, b_1_ is the adsorption heat (cal), k_t_ is the equilibrium binding constant (L/g) corresponding to maximum binding energy. By plotting q_e_ vs. ln C_e_ one can calculate B_1_ and k_t_ from the slope and the intercept of the curve, respectively. For isotherm study, about 0.5 gram of adsorbent was added to 250 mL of dye solution at different initial dye concentrations. Experiments were carried out at 20°C and 200 rpm for 24 h to ensure achieving equilibrium.

### Thermodynamic study

The equilibrium constant k (q_e_/C_e_) was used for the determination of thermodynamic parameters such as enthalpy change (∆H^0^), entropy change (∆S^0^) and free energy change (∆G^0^). The latter was determined using the following equation:

(10) $$\Delta G^{0}=-{\mathrm{RT ln k}}_{d}$$

Where, ∆G^0^ is the free energy change of adsorption reaction, R is the universal gas constant (8.314 J/mol.K) and T is the temperature (0K). The free energy change gives the degree of spontaneity of the adsorption process. A high negative value of such parameter shows an energetically favorable adsorption. On the other hand, the values of enthalpy change (∆H^0^) and entropy change (∆S^0^) can be deduced from the following equation:

(11) $${\mathrm{ln k}}_{d}=\left(\Delta S^{0}/R\right)-\left(\Delta H^{0}/\mathrm{RT}\right)$$

The values of ∆H^0^ and ∆S^0^ can be calculated from slope and intercept of the linear plot ln k_d_ versus 1/T.

### Mass transfer coefficient

In adsorption process there are two basic mass transfer resistances named as external diffusion and internal diffusion. The external mass transfer is expressed as:

(12) $$N_{t}=k_{\mathrm{mtc}}A\left(C_{t}-C_{s}\right)$$

Where N_t_ is the diffusion rate through the film layer around the adsorbent particle, A is the external surface of adsorbent, C_s_ is the adsorbate concentration at equilibrium time, C_t_ is the adsorbate concentration at time t and k_mtc_is the external mass transfer coefficient. A reverse value of k_mtc_ indicates a resistance in the film layer. Based on mass balance equation, diffusion rate can be expressed as:

(13) $$N_{t}={\mathrm{VdC}}_{t}/\mathrm{dt}={\mathrm{mdq}}_{t}/\mathrm{dt}$$

At initial condition, Eq ((13)) can be rearranged as follows:

(14) $${\left(N_{t}\right)}_{t}\to 0=k_{\mathrm{mtc}}{\mathrm{AC}}_{0}$$

Using the initial shape of pseudo-second order kinetic model, the following equation can be deduced from Eq (13) and Eq (14):

(15) $$k_{\mathrm{mct}}={{\mathrm{mkq}}_{e}}^{2}/\left(C_{0}A\right)$$

Eq. (15) allows calculating external mass transfer coefficient, k_mct_[23].

### Desorption experiments

Desorption experiments were conducted to assess the reusability of the used pumice. Two different desorption methods were therefore investigated, the pH and the heating methods. In the heating method, the pumice was added onto 250 mL dye solution (160 mg/L dye) and let until reaching equilibrium. The removal percentage was then calculated. After that the pumice stone was extracted, rinsed with distilled water for several times and heated at 150°C for 6 hours. The pumice stone from this step was added again to 250 mL dye solution (160 mg/L) and let until reaching equilibrium. By comparison of removal percentages before and after heating, desorption yield was determined. In the case of the pH method, the used adsorbent was introduced into 250 mL beaker and then NaOH at various normalities was added. The beaker was shaken at 200 rpm for 30 min and then the desorbed adsorbent was taken, dried and used again to investigate what percentage of adsorbent was regenerated.

## Results

**Figure 1:**
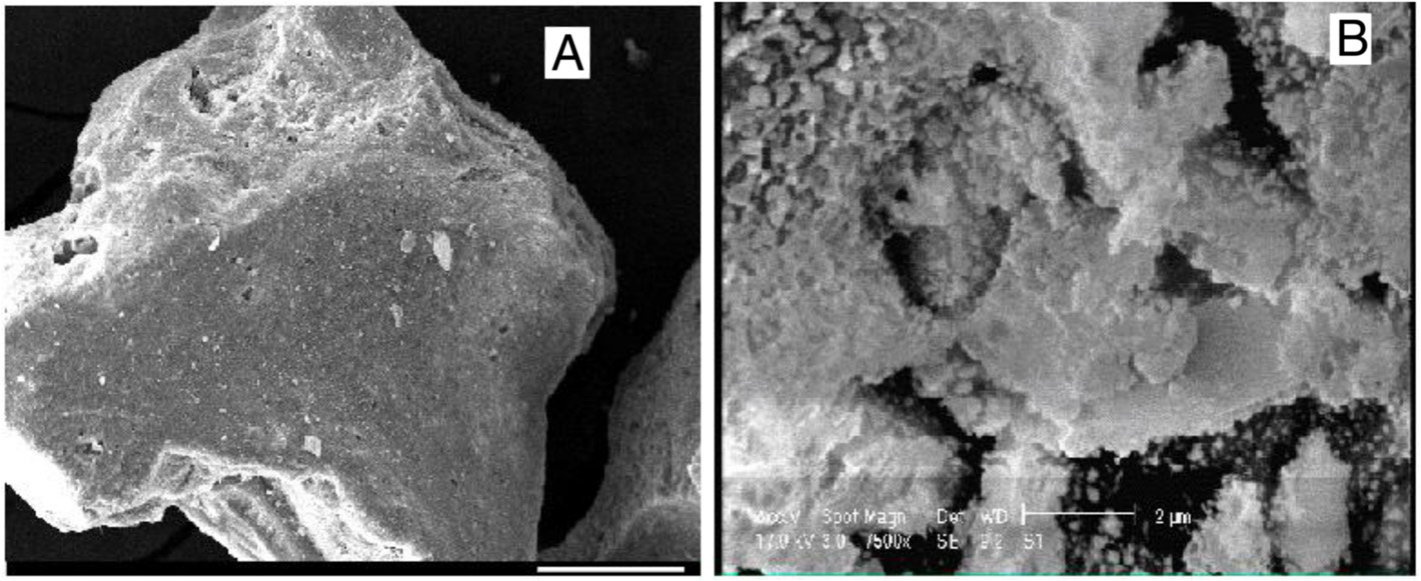
The SEM image of the adsorbent before (A) and after (B) modification.

**Figure 2:**
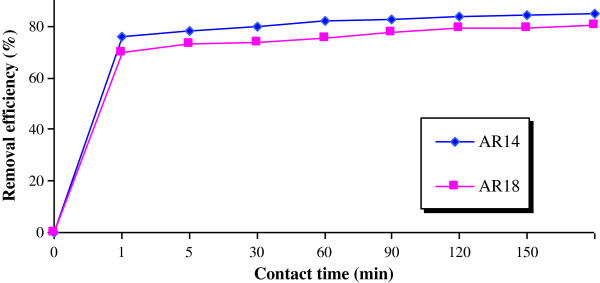
Effect of the contact time on dye removal (pH=3.5, dye concentration=100 mg/L, pumice=0.5 g).

**Figure 3:**
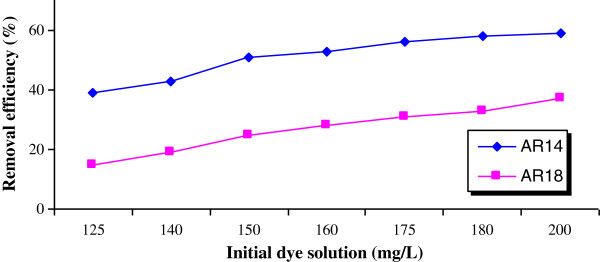
Effect of the initial dye concentration on dye removal (contact time=180 min, pH=3.5, pumice=0.5 g).

**Figure 4:**
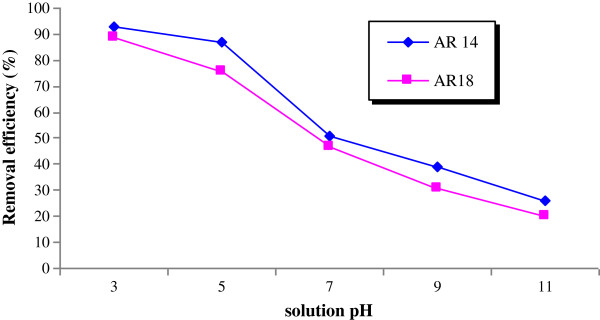
Effect of pH on Dye removal (pumice=0.5 g, dye concentration=140 mg/L, contact time=180 min).

**Figure 5:**
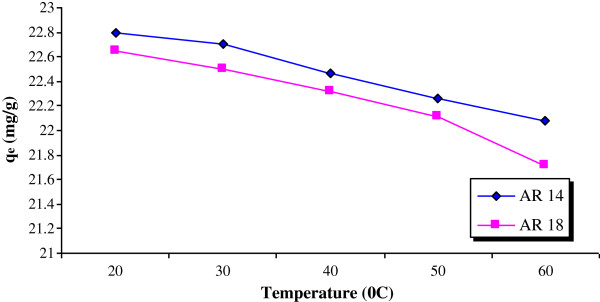
Effect of the temperature on dye removal (pumice=0.5 g, pH=3.5, initial dye concentration= 100 mg/L).

**Figure 6:**
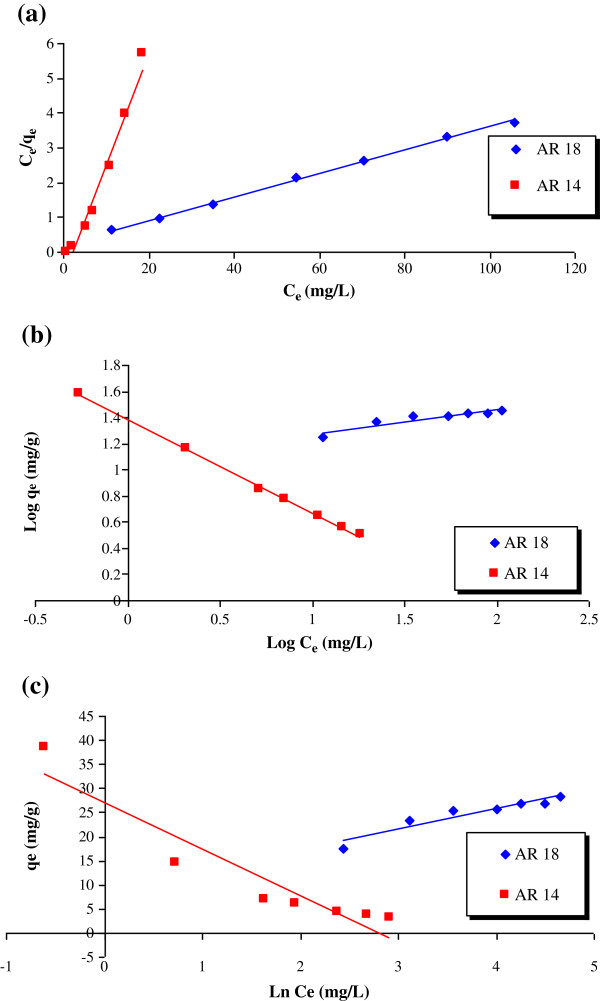
Fitting of experimental data onto isotherm model (a) Langmuir model, (b) Freundlich model, (c) Temkin isotherm model.

**Figure 7:**
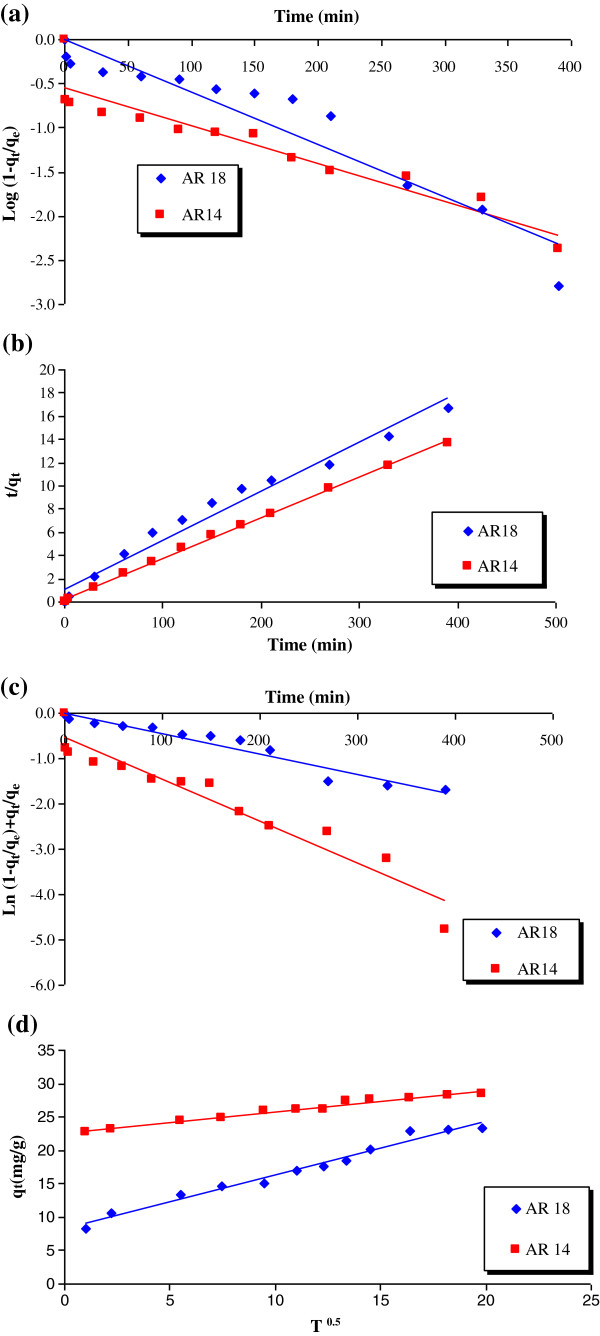
Fitting of experimental data onto kinetic model, (a) pseudo-first order, (b) pseudo-second order, (c) modified pseudo-first order, (d) intra-particle diffusion model.

**Figure 8:**
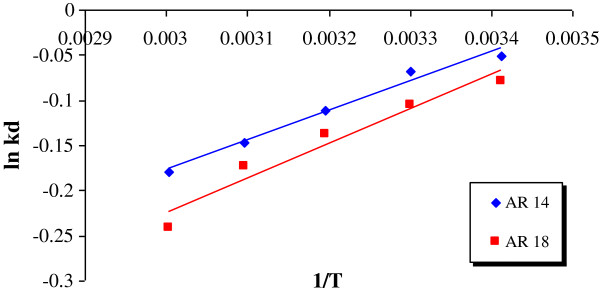
Thermodynamic parameters for the adsorption of AR14 and AR18 on pumice stone (C0=100 mg/L, pH=3.5, contact time=60 min).

Figure 1 shows the SEM image for the adsorbent before and after modification. Table 2 shows the chemical composition of the adsorbent after treat with acid. Figure 2 shows high initial rate of removal, which then decreased abruptly after only 1 minute of contact time. After 1 min, 75% and 70% of AR14 and AR18 were removed, showing the high capacity of pumice stone for dye removal. Removal of dyes increased for increasing in initial dye concentration (Figure 3.). By increasing of initial dye concentration, removal efficiency was increased from 39% and 15% to 59% and 37% for AR 14 and AR18, respectively. The effect of pH on dye removal is displayed in Figure 4 and showing a decrease of the removal for both dyes with increasing pH, from about 99% to 25% at pH=3 and 11, respectively. The effect of solution temperature is shown in Figure 5. Adsorption of both dyes decreased with increasing solution temperatures. Figure 6 shows the isotherm model and the related parameters are given in Table 3. Analysis of linear regression shows that removal of AR18 and AR14 followed Langmuir (r^2^>0.99) and Freundlich (r^2^>0.99) isotherm mo dels, respectively. The calculated *R*_*L*_ values at different initial dye solutions for AR14 and AR18 was in the order of magnitude of 0.028-0.01 and 0.093-0.034, respectively, which showed a favorable adsorption since for both dyes the values were between 0 and 1 (according to Table 2). The results of kinetic study are shown in Figure 7 and the corresponding parameters in Table 4. Figure 8 shows the results of the thermodynamic studies. External mass transfer coefficients at various initial dye concentrations are listed in Table 5, showing decreases in external mass transfer coefficients with increase in initial dye concentration. The overall results show that dye removal increased with increases in contact time and initial dye concentration, while decreased with increase in pH and solution temperature.

**Table 3:** Parameters collected from isotherm models

	**q**_**m**_**(mg/g)**	**b(L/mg)**	**K**_**f**_	**n**	**b**_**1**_	**k**_**t**_	**R**^**2**^
AR18							
Freundlich	----------	---------	12.17	5.4	----	-----	0.87
Langmuir	29.7	0.14	------	------	----	-----	0.9983
Temkin	----------	---------	------	------	0.6	8.24	0.9
AR14							
Freundlich	-----------	-----------	24.2	1.4	---	---	0.9975
Langmuir	3.125	0.5	------	------	---	----	0.9699
Temkin	---------	-----------	-----	----	0.3	17	0.88

**Table 4:** Parameters extracted from kinetic models

**AR18**	**k**_**1**_	**k**_**2**_	**k**_**m**_	**k**_**i**_	**q**_**e(calcu)**_	**q**_**e(actu)**_	**R**^**2**^
Pseudo-first order	0.006	-------	----------	-----	23.32	23	0.90
Pseudo-second order	--------	0.002	---------	-----	23.75	23.75	0.97
Modified pseudo-first order	--------	-------	0.0045	-----	25.1	23	0.95
Intraparticle diffusion	------	------	----------	0.8	-------	-----	0.98
AR 14							
	k_1_	k_2_	k_m_	k_i_	q_e(calcu)_	q_e(actu)_	R^2^
Pseudo-first order	0.004	-------	----------	-----	28.58	28	0.88
Pseudo-second order	---------	0.007	----------	-----	28.4	28.4	0.99
Modified pseudo-first order	---------	-------	0.0092	-----	28.55	28	0.93
Intraparticle diffusion	---------	-------	---------	0.3	--------	-------	0.97

**Table 5:** Mass transfer coefficients for various initial dye concentrations

**AR18**							
C_0_ (mg/L)	70	100	120	140	160	180	200
K_mct_ (cm/s)	0.0075	0.0091	0.0092	0.0079	0.0076	0.0069	0.0067
**AR14**							
C_0_ (mg/L)	70	100	120	140	160	180	200
K_mct_ (cm/s)	0.043	0.0042	0.0008	0.0005	0.0002	0.00015	0.0001

## Discussion

Adsorption mechanism of used azo dyes can be easily described from the examination of the chemical composition of adsorbent and dyes. As shown in Table 1, the main component of the sorbent was SiO_2_ (51.45%). According to the following equation, in aqueous solution this component can discompose to Si^+2^ and O_2_^-2^ at the surface of the adsorbent, leading to the formation of Si^+2^ species at the surface of the adsorbent:

(16) $${\mathrm{SiO}}_{2}↔{\mathrm{Si}}^{+2}+{O_{2}}^{-2}$$

In addition, in aqueous solution acid dyes are first dissolved and then are discomposed to the sulfonate groups of acid dye and hence to the anionic dye group as follows:

(17) $${\mathrm{DSO}}_{3}\mathrm{Na}\to {{\mathrm{DSO}}_{3}}^{-}+{\mathrm{Na}}^{+}$$

In light of Eq. ((16)) and Eq. ((17)), the adsorption process continued due to electro-static interaction of DSO_3_^-^ and Si^+2^ as two counter groups:

(18) $${\mathrm{Si}}^{+2}+{{\mathrm{DSO}}_{3}}^{-}↔\mathrm{Si}{\left({\mathrm{DSO}}_{3}\right)}_{2}$$

Moreover, in the presence of protons, they are added to the surface of the adsorbent increasing the cationic capacity of the adsorbent [15,16]:

(19) $$H^{+}+{\mathrm{Si}}^{+2}↔{\mathrm{His}}^{+3}$$

From the above equation, the sorption capacity of pumice in acidic condition would increase. Differences in removal yields between dyes can be ascribed to the molecular weight of dyes, their chemical structure and the number of sulfonate groups. AR18 has a high molecular weight in comparison with AR14. The lower molecular weight of AR14 allowed a more efficient penetration in the internal part of the adsorbent, leading to a higher removal yield than AR18. In addition, a lower number of sulfonate groups as was the case for AR 14 if compared to AR 18 resulted in a lower size and a lower strict hindrance and hence the penetration in the adsorbent was easier. These phenomena can be considered to account for the results obtained from equilibrium study and desorption experiment as seen below.

As shown on Figure 1 and Table 2, the pumice adsorbent showed an irregular structure after modification. The irregular structure is responsible for increasing in dye sorption capacity. In addition, pumice stone shows smaller pore structure after treatment by HCL. The initial specific surface of pumice stone was 28 m^2^/g and increased to 54 m^2^/g after acidic treatment.

AR14 has a low molecular weight and hence can penetrate into the internal part of pumice adsorbent, in agreement with previous results recorded using fly ash as adsorbent [14]. In addition, previous results recorded using activated carbon prepared from Poplar wood [3], which displayed a high specific surface area (385m^2^/g) but a low porosity, showed lower yield and rate of removal of AR18, if compared to the present results obtained on pumice stone. It should be most likely related to the high porosity of pumice stone, showing adsorption not only at the surface, but also in the internal part of the sorbent.

Removal yields increased almost linearly for increasing initial dye concentrations (Figure 3), which can most likely be attributed to increasing driving force for increasing initial dye concentrations [22]. Irrespective of the initial dye concentration, higher removal yields were recorded for AR14, if compared to AR18. This phenomenon can be described by large number of mere sites for adsorption of dye at initial stage and with time elapse, and owing to the repulsive forces between sorbate and bulk phase, the occupation of the remaining sites became more difficult to dye molecules [22]. In addition and based on isotherm study described thereafter, due to the higher affinity of AR14 if compared to AR18, higher removal rates were observed for the former at initial sorption stage.

The pH has an important role in the adsorption process. According to the pH, the surface of the adsorbent will be positively or negatively charged. As the pH_zpc_ of pumice was 7.2, at higher and lower values the surface of pumice will be occupied by OH^-^ and H^+^ ions, respectively. When introduced into the solution, Azo dyes dissolved and formed negative sulfonate groups. Due to the positive charge of pumice in acidic conditions, the following reaction would occur:

(20) $$M-H^{+}+{{\mathrm{DSO}}_{3}}^{-}\to M-{\mathrm{HDSO}}_{3}$$

Therefore, acidic conditions leading to increasing amount of H^+^ ions, especially at the surface of pumice, consequently improve dye removal [15,16].

A low temperature was therefore more suitable for AR14 and AR18 removal, showing that the adsorption was exothermic in nature. An escape of dye molecules from the solid surface to the solution for an increment in temperature can account for this behavior.

Fitting of obtained data onto three isotherm model show the removal of AR 14 and AR 18 follow Freundlich (r^2^>0.99) and Langmuir (r^2^>0.99) isotherm model. Maximum sorption capacities were 29.7 and 3.125 mg/g for AR 18 and AR 18, respectively; according to Langmuir isotherm constant. The high value of K_f_ was observed for AR 14 rather than AR 18, showing high affinity of AR 14 onto pumice stone. The value of Freundlich constant (n) was 5.4 and 1.4 for AR 18 and AR 14, respectively; showing the favorable nature of present sorption process. Removal of AR14 by three soils, namely GSE17200, GSE1201 and DG06 has been studied by Baocheng *et al.*[5], and showed that AR14 removal followed a Freundlich isotherm model. Maximum capacities of DG06, GSE17200 and GSE17201 were 1.3, 0.98 and 0.83 (mg/g) respectively, namely below that of pumice for AR14 (3.125 mg/g). AR18 removal was previously investigated using activated carbon derived from Poplar wood [3]. The K_f_ value and maximum sorption capacity were 1.5 and 3.9 (mg/g) respectively, namely significantly lower than those obtained in this work using pumice stone, 12.17 and 29.7 (mg/g) respectively.

It is clear from Table 4 that removal of AR18 and AR14 followed Intra-particle diffusion (r^2^>0.98) and pseudo-second order (r^2^>0.99) kinetic model, respectively. However, regression of intra-particle diffusion model did not pass through the origin, showing that it was not the rate-determining step. It can also be observed, that for both dyes similar values were observed for calculated and experimental q_e_.

The values of ∆H^0^ and ∆S^0^ for AR14 were found to be −2698 (kJ/mol) and −9.56 (kJ/mol.K), respectively; while for AR18 they were −3165.88 (kJ/mol) and −11.35 (kJ/mol.K), respectively. The negative value of enthalpy change (∆H^0^) for both dyes demonstrated that adsorption of AR14 and AR18 onto the present medium was exothermic in nature. The negative values of ∆S^0^ for both dyes indicated a decrease in the fortuitousness of adsorption at the solid/liquid interface. On the other hand, Gibbs free energy (∆G^0^) for AR14 and AR18 were found to be in the ranges 124.9- 494.7 and 191.3-667.4 for temperatures in the range 293–333 K, respectively. The positive values of the Gibbs free energy for both dyes indicated that sorption of AR14 and AR18 onto acidic treated pumice were not thermodynamically spontaneous.

For both dyes the values of external mass transfer coefficient decreased for increasing initial dye concentrations. Increasing values of k_mtc_ can be related to increasing external mass transfer resistance at solid/liquid layer. The result of external mass transfer coefficient for both dyes was in the order of 10^-3^-10^-4^, in agreement with values of order of magnitude 10^-4^ found in the literature [23,24].

Desorption experiments are needed to investigate the economic feature of adsorption. As shown in Figure 4, dye removal was low at high pH, which demonstrated that desorption can be done at alkaline pH. Heating the used adsorbent is another way to perform desorption. In this work, both methods were investigated. By heating method, about 63% and 76% regeneration was observed for AR14 and AR18, respectively. High regeneration of pumice for AR18 can be ascribed to the low affinity of AR18 (K_f_=12.17) onto pumice surface and the release of SO_3_^-^ from the surface of the adsorbent. By pH method, the adsorbed regeneration percentages were 64%, 72% and 86% for AR14 and were 65%, 71% and 89% for AR18 for 1, 1.5 and 2 N NaOH, respectively. Resulting from pH increase, the surface of the adsorbent bombed by OH^-^ ions led to the extraction of SO_3_^-^ and H^+^ from the surface of the adsorbent. In addition, high OH^-^ ion concentrations increased the driving force of others ions to be adsorbed on the surface of the medium.

From the above comments, it can be concluded that regeneration of pumice can be efficiently done by the heating method.

## Competing interests

The authors declare that they have no competing interests.

## Authors’ contribution

MRS was involved in the experimental part of the work, MZ the first contributor of this work, was involved in all experiments presented in this paper and also writes the first draft of the paper, MNS was involved in the work plan and in the discussion of the results, AA read and corrected the manuscript, GHS was involved in the discussion of the results and read the manuscript and SB was involved in the discussion of the results and read the manuscript. All authors read and approved the final manuscript.
